# 32Starch metabolism in potato Solanum tuberosum L.

**DOI:** 10.18699/VJGB-22-32

**Published:** 2022-05

**Authors:** E.M. Sergeeva, K.T. Larichev, E.A. Salina,, A.V. Kochetov

**Affiliations:** Institute of Cytology and Genetics of the Siberian Branch of the Russian Academy of Sciences, Novosibirsk, Russia; Institute of Cytology and Genetics of the Siberian Branch of the Russian Academy of Sciences, Novosibirsk, Russia; Institute of Cytology and Genetics of the Siberian Branch of the Russian Academy of Sciences, Novosibirsk, Russia; Institute of Cytology and Genetics of the Siberian Branch of the Russian Academy of Sciences, Novosibirsk, Russia

**Keywords:** potato, Solanum tuberosum, starch, amylose, amylopectin, synthesis, degradation, картофель, Solanum tuberosum, крахмал, амилоза, амилопектин, синтез, деградация

## Abstract

Starch is a major storage carbohydrate in plants. It is an important source of calories in the human and animal diet. Also, it is widely used in various industries. Native starch consists of water-insoluble semicrystalline granules formed by natural glucose polymers amylose and amylopectin. The physicochemical properties of starch are determined by the amylose:amylopectin ratio in the granule and degrees of their polymerization and phosphorylation. Potato Solanum tuberosum L. is one of the main starch-producing crops. Growing industrial needs necessitate the breeding of plant varieties with increased starch content and specified starch properties. This task demands detailed information on starch metabolism in the producing plant. It is a complex process, requiring the orchestrated work of many enzymes, transporter and targeting proteins, transcription factors, and other regulators. Two types of starch are recognized with regard to their biological functions. Transitory starch is synthesized in chloroplasts of photosynthetic organs and degraded in the absence of light, providing carbohydrates for cell needs. Storage starch is synthesized and stored in amyloplasts of storage organs: grains and tubers. The main enzymatic reactions of starch biosynthesis and degradation, as well as carbohydrate transport and metabolism, are well known in the case of transitory starch of the model plant Arabidopsis thaliana. Less is known about features of starch metabolism in storage organs, in particular, potato tubers. Several issues remain obscure: the roles of enzyme isoforms and different regulatory factors in tissues at various plant developmental stages and under different environmental conditions; alternative enzymatic processes; targeting and transport proteins. In this review, the key enzymatic reactions of plant carbohydrate metabolism, transitory and storage starch biosynthesis, and starch degradation are discussed, and features specific for potato are outlined. Attention is also paid to the known regulatory factors affecting starch metabolism

## Abstract (ru)

Крахмал – основной запасной углевод растений и важный источник калорий в рационе питания человека и животных, широко применяется также для ряда промышленных нужд. Нативный крахмал представляет собой не растворимые в воде полукристаллические гранулы, сформированные молекулами амилозы и амилопектина, которые являются природными полимерами глюкозы. Физико-химические свойства крахмала определяются соотношением амилозы и амилопектина в грануле, степенью их полимеризации и фосфорилирования. Одно из основных растений-продуцентов крахмала – картофель Solanum tuberosum L. Растущие потребности рынка диктуют необходимость получения сортов с повышенным содержанием и заданными свойствами крахмала, необходимым условием чего является получение детальной информации о процессе его метаболизма в организме растения. Процесс метаболизма крахмала сложен и представляет собой согласованную работу множества ферментов, транспортных и направляющих белков, транскрипционных и других регуляторных факторов. По принципу выполняемой биологической функции крахмал делится на два типа: транзиторный, который синтезируется в хлоропластах фотосинтезирующих органов и распадается при отсутствии освещения, обеспечивая клетку углеводами; и запасной, который синтезируется и хранится в амилопластах запасающих органов (зерен, клубней). Основные ферментативные реакции биосинтеза и деградации крахмала, а также транспорта и метаболизма углеводов хорошо изучены на транзиторном крахмале модельного объекта Arabidopsis thaliana; об особенностях метаболизма крахмала в запасающих органах, в частности клубнях картофеля, известно несколько меньше. При этом ряд вопросов остается открытым: недостаточно изучены участие различных изоформ ферментов и влияние на них регуляторных факторов в зависимости от ткани и стадии развития растения, а также условий внешней среды; реализация альтернативных путей ферментативных превращений; участие направляющих и транспортных белков. В данном обзоре рассмотрены ключевые ферментативные реакции углеводного обмена, биосинтеза и деградации транзиторного и запасного крахмала, общие для растений, и отмечены особенности, характерные для картофеля; уделено внимание известным регуляторным факторам, влияющим на метаболизм крахмала.

## Introduction

Starch is the main storage carbohydrate in plants. It constitutes up to 85 % of the dry matter of their edible parts: cereal grains (maize Zea mays L., rice Oryza sativa L., wheat Triticum spp., barley Hordeum vulgare L., etc.), potato tubers Solanum tuberosum L., edible roots (cassava Manihot esculenta Crantz, sweet potato Ipomoea batatas (L.) Lam., and yam Dioscorea alata L.), sago palm stems Metroxylon sagu Rottb., plantain fruit Musa spp. (Zeeman et al., 2010; Santana, Meireles, 2014). Starch provides a great portion of calories for human and animal nutrition. In addition, it is a natural reproducible and biodegradable material used in nonfood industry, e. g., in the production of fabric, paper, drugs, and plastics

Chemically, starch is a mixture of amylose and amylopectin. These natural glucose polymers form waterinsoluble semicrystalline granules. Amylopectin consists of highly branched glucan molecules, the linear regions of which are formed by α-1,4-glycosidic bonds, whereas the branching points are formed by α-1,6-bonds. Amylose is a practically linear polymer with few branches. Amylopectin molecules constitute about 75–80 % of starch weight. They form the structural framework of the granule, consisting of repetitive amorphous and semicrystalline lamellae. Amylose molecules are dispersed in the semicrystalline amylopectin matrix (Zeeman et al., 2010; Tetlow, Bertoft, 2020).

The amylose:amylopectin ratio determines the dietetic, physicochemical, and functional starch properties essential for particular industries. The starch present in food is classified into glycemic and resistant. Being readily digestible in the small intestine, amylopectin increases the glycemic potential of starch. In contrast, higher amylose contents make starch more resistant (Li et al., 2008). Resistant starch is less degradable by amylases in the small intestine. It serves as substrate for microbes in the large intestine to produce short-chain fatty acids, which exert local antiinflammatory and antitumor effects (Birt et al., 2013). Also, physicochemical (gelatinization and retrogradation) and functional (swelling and viscosity) properties are taken into consideration in certain applications. These properties are determined genetically: by size and morphology of starch granules, amylose:amylopectin ratio, glucan branching, and glucan phosphorylation (Visser et al., 1991; Schwall et al., 2000; Hofvander et al., 2004; Khlestkin et al., 2017).

Potato (Solanum tuberosum L.) ranks fourth among starch-producing crops in the world, next to maize, cassava, and wheat. Potato starch differs from cereal starches in a variety of important features. Potato amylose and amylopectin have higher degrees of polymerization and phosphorylation; therefore, potato starch is more suitable for bioplastic production (Hofvander et al., 2004; Reyniers et al., 2020). In response to the increasing commercial demand, the global production of potato starch steadily increases: 3.7 million tons in 2018 and 3.9 million tons in 2020 (https://www.researchand markets.com/reports/5330932/potato-starch-marketglobal- industry-trends). To obtain native starches with specified properties and to increase the overall amount of starch per plant are topical tasks in potato breeding.

The key enzymes in starch biosynthesis (see the Table) and their genes have been studied in detail in model plants (Arabidopsis thaliana L.) and in crops, including potato (Streb, Zeeman, 2012; Van Harsselaar et al., 2017; Slugina, Kochieva, 2018).

**Table 1.**
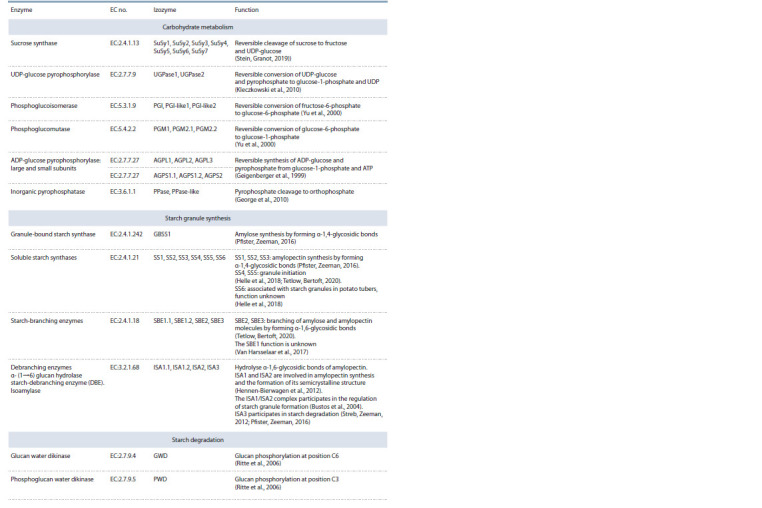
Enzymes involved in starch metabolism

**Table 2.**
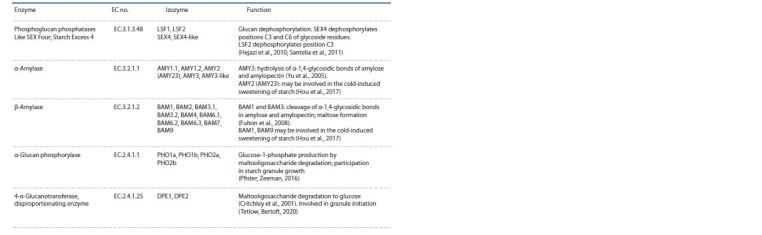
End of the Table

Starch is produced by the polymerization of ADPglucose, catalyzed by granule-bound (GBSS) and soluble (SS) starch synthases. Other enzymes involved are the starch branching enzyme (SBE) and the debranching enzyme (DBE).

Starch metabolism is a component of general carbohydrate metabolism. It is essential for plant functions: growth, development, and stress response. The enzymes and genes associated with the metabolism of starch and other plant carbohydrates have been extensively studied for three decades. A considerable body of data on the location of these genes in plant genomes and their expression has been accumulated. Seventy-five S. tuberosum genes have been mapped on the reference genome, and the expression patterns of 64 genes in leaves and tubers have been studied (Van Harsselaar et al., 2017; Slugina, Kochieva, 2018). It has been shown that starch metabolism genes experienced numerous duplications and produced paralogs by sub- and neofunctionalization during evolution. The paralogous genes, which encode different enzyme isoforms, show tissue- and/or stage-specific expression patterns (Van Harsselaar et al., 2017; Qu et al., 2018; López-González et al., 2019). Various factors affect the expression of starch metabolism genes: circadian rhythms, photoperiod, levels of plant hormones and sugars, and stressing factors (drought and cold) (López-González et al., 2019). Although the key enzymatic reactions in starch biosynthesis and degradation are well known, there are many unclear points concerning alternative enzymatic processes and their localization inside the cell, roles of particular isozymes in starch metabolism in different organs at different developmental stages, and the influence of regulatory factors.

By now, functions of many proteins involved in starch metabolism in potato have been identified (see the Table). Most enzymes have isoforms with partially overlapping functions (Van Harsselaar et al., 2017). Translocator proteins essential for transporting metabolites through plastid membranes are important for starch metabolism as well. They also have multiple forms: adenylate translocators NTT1 and NTT2 (Tjaden et al., 2001), glucose transporter pGlcT1 (Cho et al., 2011), glucose 6-phosphate translocator (isoforms GPT1.1, GPT1.2, GPT2.1, and GPT2.2) (Kammerer et al., 1998), maltose transporter MEX1 (Cho et al., 2011), and triose phosphate translocator (TPT, TPT-like) (Flügge et al., 1989).

The amylose:amylopectin ratio in plants can be modified by raising lines carrying certain alleles of genes involved in starch synthesis. Such accessions were obtained in cereals; for instance, in maize Z. mays. The amylose extender (ae−) mutation is associated with the loss of the activity of the starch-branching enzyme SBEIIb. Starch in plants with the ae− phenotype is enriched with amylose, and its amylopectin chains are longer (Stinard et al., 1993). The maize phenotype whose starch has practically no amylose is named waxy, and it is determined by a mutation in the gene for granulebound starch synthase GBSSI. Its endosperm is gluey (Hossain et al., 2019).

However, the breeding of potato varieties with specified starch properties is complicated by its autotetraploid genome. The only amylose-free potato cultivar Eliane was obtained by mutation-assisted breeding (Muth et al., 2008). Genetic engineering and genome editing aimed at the modification of key starch biosynthesis genes produced plants with expected phenotypes: amylose-free starch (knockout and knockdown of the GBSS1 gene), amylose-enriched starch (knockdown of both SBE1 and SBE2), and starch with modified amylopectin properties (editing of SBE1 and/or SBE2) (Visser et al., 1991; Schwall et al., 2000; Hofvander et al., 2004; Andersson et al., 2006, 2017; Tuncel et al., 2019).

In some cases, plants with a desired phenotype acquired additional traits. For example, the increase in amylose content obtained by antisense suppression of the genes for starch-branching enzymes SBE1 and SBE2 was accompanied by a decrease in starch content, formation of smaller granules, and larger tubers (Hofvander et al., 2004). Apparently, changes in certain starch metabolism steps may affect the overall carbohydrate metabolism in the plant. An association study revealed genetic loci associated with starch content and productivity (tuber weight), whereas the functions of some detected genes were unknown at all, and some other genes were involved in signaling and regulation: transcriptional and posttranscriptional (Schönhals et al., 2017). Part of the detected SNPs exerted antagonistic effects on potato productivity and starch content (Schönhals et al., 2017). Thus, the investigation of regulation pathways of starch metabolism genes is important for improving potato quality and productivity.

## Carbohydrate metabolism in potato plants

Two starch forms are recognized with regard to biologic function: transitory and storage. Transitory starch is synthesized and accumulated in chloroplasts of photosynthetic organs (leaves) in the daytime and degraded in darkness to provide nutrients for the cell. Storage starch is synthesized in amyloplasts (nonphotosynthetic plastids) of storage organs (e. g., potato tubers) and stored there for a long time to be utilized in the preparation for sprouting (Zeeman et al., 2010; Streb, Zeeman, 2012).

The main difference in carbohydrate metabolism between cells of leaves and storage organs is in the sources of carbohydrates and ATP required for starch synthesis and enzymatic reactions. In leaves, they can form in the same cells that produce transitory starch, and in storage organs, they are imported from photosynthetic ones.

During photosynthesis, chloroplasts produce ATP and fix atmospheric carbon dioxide by the Calvin–Benson cycle, in which triose phosphate is produced as an intermediate (Streb, Zeeman, 2012). Part of triose phosphate molecules remain in the chloroplast stroma to serve as carbohydrate material for transitory starch synthesis. The rest is transported to cytosol by triose phosphate translocator TPT (Flügge et al., 1989). In chloroplasts, a series of enzymatic reactions converts triose phosphate to glucose-1-phosphate (G1P). Then ADP-glucose pyrophosphorylase (AGPase) converts G1P to ADPglucose, the main substrate for starch synthesis (see the Figure).

**Fig. 1.**
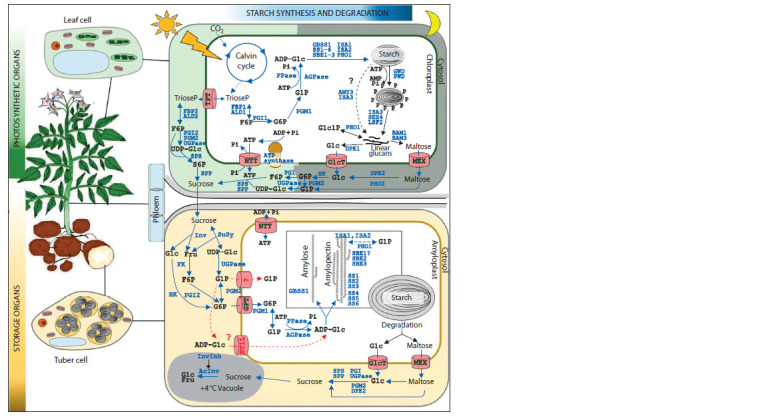
Starch metabolism in potato photosynthesizing (leaves) and storage (tubers) organs. Blue arrows indicate the starch biosynthesis and sugar metabolism pathways; black arrows, the starch degradation pathway. Starch degradation in leaves and starch biosynthesis in tubers are shown in more detail. The red arrow indicates the alternative pathway of ADP-glucose synthesis in cytosol and subsequent transport to amyloplasts performed by BT1 (Brittle1-like transporter). Carbohydrates are shown in black: Fru, fructose; F6P, fructose-6-phosphate; Glc, glucose; G1P, glucose-1-phosphate; G6P, glucose-6-phosphate; S6P, sucrose-6-phosphate; TrioseP, triose phosphate; ADP-Glc, ADP-glucose; UDP-Glc, UDP-glucose. Enzymes and invertase inhibitor (in blue): AcInv, vacuolar acid invertase; AGPase, ADP-glucose pyrophosphorylase; ALD, aldolase; AMY, α-amylase; BAM, β-amylase; FK, fructokinase; DPE, 4-α-glucanotransferase; FBP, fructose-1,6-bisphosphatase; GBSS, granule-bound starch synthase; GWD, α-glucan water dikinase; HK, hexokinase; ISA, isoamylase; InvInh, invertase inhibitor; Inv, invertase; PGI, phosphoglucoisomerase; PGM, phosphoglucomutase; PHO, α-glucan phosphorylase; PPase, inorganic pyrophosphatase; PWD, phosphoglucan water dikinase; SBE, starch-branching enzyme; SEX, LSF, phosphoglucan phosphatases; SPP, sucrose phosphate phosphatase; SPS, sucrose phosphate synthase; SS, starch synthase; SuSy, sucrose synthase; UGPase, UDP-glucose pyrophosphorylase. Transporter proteins (in green): GPT, glucose-6-phosphate translocator; NTT, ATP-ADP antiporter; MEX, maltose transporter; TPT, triose phosphate/phosphate translocator; GlcT, glucose transporter; BT1L, Brittle1-like transporter.

As triose phosphate molecules are exported to cytosol, they are converted to sucrose, which is then delivered to storage organs through phloem and apoplast to be a carbohydrate material for storage starch synthesis. Sucrose is transported into cells of storage organs either by sucrose transporter proteins or, after being hydrolyzed by invertase to glucose and fructose, by hexose transporters (Ruan, 2014). There are two pathways to cleave sucrose in cell cytoplasm: saccharolytic, catalyzed by sucrose synthase SuSy, or hydrolytic, catalyzed by invertase Inv. Invertase irreversibly cleaves sucrose to glucose and fructose, and SuSy catalyzes the reversible cleavage to fructose and UDP-glucose (Stein, Granot, 2019). The predominant pathway depends on the tuber development stage. At the beginning of growth, at a high cell division rate, the hydrolytic pathway prevails, and the saccharolytic pathway steps forward at the starch accumulation stage (Appeldoorn et al., 1997). The SuSy-catalyzed pathway is important for the rate of starch accumulation in potato. It has been shown that a decrease in SuSy activity reduces starch content in mature tubers (Zrenner et al., 1995; Baroja-Fernández et al., 2009). Seven SuSy isoforms have been predicted in potato, and the SuSy4 gene had tissue-specific expression in growing tubers (Van Harsselaar et al., 2017). It is likely that some sucrose synthase isoforms are involved in sucrose transport through phloem, as observed in A. thaliana (Yao et al., 2020).

The subsequent steps, catalyzed by fructokinase FK, hexokinase HK, and UDP-glucose pyrophosphorylase, produce a pool of phosphorylated hexoses in cytosol. They can be reversibly interconverted by cytosolic isoforms of phosphoglucoisomerase PGI2 and phosphoglucomutase PGM2 (Yu et al., 2000; Kleczkowski et al., 2010). One of the phosphorylated hexoses, glucose- 6-phosphate, is transported to amyloplasts by membrane glucose-6-phosphate/phosphate-translocator GPT (Kammerer et al., 1998). In amyloplasts, G6P is utilized for ADP-glucose production. The rate of starch biosynthesis in potato tubers directly depends on G6P transport (Tauberger et al., 2000; Fernie et al., 2002).

ATP is also imported to storage tissues from photosynthetic ones. It is delivered inside amyloplasts by plastid adenylate translocator NTT. It is known that even a slight decrease in NTT activity reduces the overall starch content in potato tubers (Tjaden et al., 2001), whereas the combination of NTT and GPT overexpression increases it (Zhang L. et al., 2008). There may be an alternative route of hexose transport to amyloplasts: direct import of glucose-1-phosphate (G1P) and its utilization in ADP-glucose synthesis. There is evidence that this route acts in potato, although the corresponding transporter protein is not known yet (Fettke et al., 2010). Two candidate G1P transporters through plasma membrane were recently found in A. thaliana (Malinova et al., 2020).

ADP-glucose acts as the substrate for starch biosynthesis. It is produced in a reversible reaction catalyzed by ADP-glucose pyrophosphorylase (AGPase) in the stroma of chloroplasts and amyloplasts. AGPase synthesizes ADP-glucose and pyrophosphate (PPi) from G1P and ATP. AGPase is a heterotetramer consisting of two large and two small subunits, AGPL and AGPS. Its activity is essential for starch synthesis in potato tubers (Geigenberger et al., 1999). Inorganic pyrophosphatase (PPase) degrades pyrophosphate to orthophosphate (George et al., 2010). The plastid PPase isoform contributes much to starch accumulation in potato tubers. Lines knocked down for the StpsPPase gene had lower contents of starch, in particular, amylose, and smaller granules. That study also recorded elevated amounts of starch biosynthesis intermediates: pyrophosphate, glucose, fructose, hexose phosphates, and, unexpectedly, ADP-glucose. The increase in ADP-glucose content indicates that pyrophosphate does not affect the direction of the AGPase-catalyzed reaction in potato. Thus, the mechanism by which PPase participates in starch synthesis in potato tubers is still to be understood (Andersson et al., 2018).

An alternative cytosolic pathway of ADP-glucose synthesis, catalyzed by SuSy and UGPase, acts in cereals (monocots). It is essential for grain growth. ADPglucose is transported to amyloplast by the Brittle1-like transporter protein (BT1) (Bowsher et al., 2007). The homolog protein of BT1 (StBT1) has been found in S. tuberosum plant. However, there is no evidence for ADPglucose transport through the amyloplast membrane. The StBT1 protein performs unidirectional transport of AMP, ADP, and ATP (Leroch et al., 2005).

## Starch granule synthesis

Transitory starch synthesis in leaf chloroplasts and storage starch synthesis in tuber amyloplasts follow basically the same route. Amylose and amylopectin synthesis is performed by 16 key enzymes belonging to the following groups: starch synthases, starch-branching enzymes, and starch-debranching enzymes (see the Table). Most enzymes exist as isoforms, the functions of which may partly overlap (Pfister, Zeeman, 2016; Van Harsselaar et al., 2017).

Starch synthases catalyze the formation of glycosidic bonds by transferring the glucose residue of ADP-glucose to the nonreducing end of the glucose polymer. They are subdivided into granule-bound (GBSS) and soluble (SS) starch synthases. The former synthesize long chains, mainly in amylose, and long chain fragments in amylopectin. The latter include a series of isoforms: SS1, SS2, SS3, SS4, SS5, and SS6. Of them, SS1, SS2, and SS3 synthesize chains of various lengths in amylopectin (Pfister, Zeeman, 2016). The SS4 isoform performs a special function among starch synthases, as it initiates starch granule formation (Tetlow, Bertoft, 2020). One or two large starch granules instead of five to seven wild-type small ones were found in A. thaliana plants with the knocked out ss4 gene (Roldán et al., 2007). The functions of SS5 and SS6 are still vague. The C end of the SS5 protein lacks the conservative fragment characteristic of starch synthases, which has catalytic domain GT1, although the protein has the conservative glucanbinding site. Probably, SS5 is involved in starch granule initiation, as it has been shown that the loss of SS5 from A. thaliana reduces the granule number in leaves (Abt et al., 2020). The SS6 isoform and its gene were found in potato in recent years (Van Harsselaar et al., 2017), and the role of this enzyme is unknown. It may participate in granule growth, as it is directly bound to it; in addition, it bears conservative motifs XXGGL and KXGGL, characteristic of glycosyl transferase domains of starch synthases GT1 and GT5, respectively (Helle et al., 2018).

Starch granule initiation was an obscure issue for a long time. The studies reported by now concern transitory starch initiation in the model plant A. thaliana, but it seems that our notion of some key steps in granule formation may be extended to other plant species (Mérida, Fettke, 2021). As mentioned above, SS4 is the main granule-initiating enzyme, and SS5 and SS6 also take part in the process. Maltooligosaccharides, probably forming in the degradation of starch polyglucans by amylases, are the substrate (Mérida, Fettke, 2021). The steric interaction of starch synthases, substrate molecules, and the growing granule is driven by the PTST2 and PTST1 proteins, targeting to starch. They are associated with starch synthases SS4 and GBSS1, respectively. Also, they contain a carbohydrate-binding domain (Seung et al., 2015, 2017). Note that PTST2 is not found in potato tubers, and this fact indicates that the starch granule initiation processes in A. thaliana and potato differ (Helle et al., 2018). A heteromultimeric complex of isoamylases ISA1 and ISA2 has been shown to influence starch granule initiation in potato tubers. By all appearances, isoamylases suppress the formation of new starch granules by disrupting the formation of soluble glucan molecules in chloroplast stroma (Bustos et al., 2004).

As the chains of amylose and amylopectin molecules are elongated by starch synthases SS1, SS2, and SS3, starch-branching enzymes SBE attach side branches to them (Pfister, Zeeman, 2016). Starch-branching enzymes cleave α-1,4-glycosidic bonds of polyglucans, synthesized by starch synthases, and attach short chains to the so-called acceptor chain by forming α-1,6-glycosidic bonds. The starch-branching enzymes of potato have three isoforms: SBE1.1, SBE1.2, SBE2, and SBE3 (formerly designated as SBE1) (see the Table) (Van Harsselaar et al., 2017). Thus, forms referred to in other papers as SBE1 and SBE2 are designated as SBE3 and SBE2 according to the notation of Van Harsselaar et al. SBE3 produces mainly long side chains, and SBE2 produces short amylopectin chains (Tetlow, Bertoft, 2020). The roles of SBE1.1 and SBE1.2 in starch production are unknown, but studies of A. thaliana demonstrate a pleiotropic effect of SBE1 on plant growth and development. Transformants overexpressing SBE1 were white-colored and low. They had a longer life cycle and produced fewer seeds than control plants (Wang X. et al., 2010). The joint action of different isoforms affects amylopectin structure. Experiments with potato plants with the knocked out genes sbe3 and/or sbe2 (designated by the experimenters as sbe1 and sbe2) demonstrate that sbe3 inactivity results in the formation of starch with longer amylopectin chains and lower branching level. The knockout of sbe2 with active sbe3 did not affect the amylopectin structure much, but the number of starch granules in potato tubers increased and size decreased (Tuncel et al., 2019).

Debranching enzymes (DBE) are another group of enzymes involved in the formation of the amylopectin structure (see the Table). They reconstruct branched glucans into easier crystallizable forms, which is essential for granule formation (Pfister, Zeeman, 2016). Debranching enzymes include isoamylases (ISA), which catalyze the hydrolysis of α-1,6-glycosidic amylopectin bonds and remove excessive branching. Potato isoamylases include isoforms ISA1, ISA2, and ISA3. The ISA1 and ISA2 proteins can form heteromultimers, capable of more efficient removal of long outer chains of amylopectin (Hussain et al., 2003). The ISA3 isoenzyme is important for starch degradation, as it cleaves short outer chains of glucans (Streb et al., 2008). Transgenic potato plants with lower expression of the isa1, isa2, and isa3 genes had significantly less starch in developing tubers, whereas the starch contents in leaves did not change. The plants also had fewer and larger granules and higher sucrose contents, probably resulting from the increase in the overall granule surface and easier access for degrading enzymes (Ferreira et al., 2017).

In addition to starch synthases, branching and debranching enzymes, the synthesis of starch granules involves α-glucan phosphorylases. Their plastid (PHO1) and cytoplasmic (PHO2) isoforms catalyze the reversible transfer of the glycosylic group of glucose-1-phosphate to the nonreducing end of the chain of an α-1,4-bound glucan (Pfister, Zeeman, 2016). The PHO2 enzyme is involved in carbohydrate metabolism in cytoplasm, and PHO1 contributes to starch synthesis and degradation in plastids. It has been shown that at lower temperatures starch synthesis in potato tubers can also follow the phosphorylase pathway with G1P as the substrate (Fettke et al., 2012).

## Starch granule degradation

Degradation is an intrinsic part of the metabolism of starch and carbohydrates in general, although it has been studied much poorer than starch biosynthesis. The degradation pathways of transitory starch have been investigated in most detail in leaves of the model plant A. thaliana. The knowledge of starch degradation in potato tubers is limited to cold-induced sweetening and sprouting. The main steps of starch degradation are the release of soluble glucan from starch granules, glucan conversion to linear forms (maltooligosaccharides), maltooligosaccharide hydrolysis to maltose, and subsequent maltose metabolism in the cell. Starch degradation is performed by a broad range of enzymes: α- and β-amylases, isoamylase, α-glucan water dikinase (GWD), phosphoglucan water dikinase (PWD), α-glucan phosphorylase, phosphoglucan phosphatase, and 4-α-glucanotransferase (see the Table).

Starch granule degradation is initiated by GWD and PWD. They phosphorylate glucans at positions C6 and C3 of glucose residues, making them more hydrophilic and allow α-, β-, and isoamylases access to them (see the Table) (Ritte et al., 2006; Streb, Zeeman, 2012). The phosphorylation by GWD seems to play the key role in starch degradation in potato tubers and leaves (Claassen et al., 1993; Orzechowski et al., 2021). Tubers of transgenic potato plants with lower expression of the StGWD gene were less prone to starch degradation at low temperatures (Lorberth et al., 1998).

The next step of starch granule degradation is glucan hydrolysis by amylases. Potato α- and β-amylases include many isoforms, and functions of some of them are not known in detail (see the Table) (Van Harsselaar et al., 2017). By extrapolating data on A. thaliana, we suppose that β-amylases BAM1 and BAM3 hydrolyze linear fragments of amylose and amylopectin, and the degradation of branched fragments demands the debranching enzyme DBE (ISA3 in potato) (Hussain et al., 2003; Fulton et al., 2008; Pfister, Zeeman, 2016). With knocked down StBAM3, starch content in potato leaves was higher than in the wild genotype (Scheidig et al., 2002). Cold-induced sweetening in potato tubers is also affected by some amylase species: α-amylase AMY2 (AMY23) and β-amylases BAM1 and BAM9. The supposed function of BAM1 and BAM9 is starch degradation in plastids, and AMY2 is likely to degrade phytoglycogen in cytosol (Hou et al., 2017). An alternative pathway of starch degradation is observed in A. thaliana. It is initiated by α-amylase AMY3, which releases linear and branched glucans from starch granules, and these glucans are then hydrolyzed by β- and isoamylases (see the Figure) (Streb et al., 2008).

Alongside starch glucan hydrolysis by amylases, the glucans are dephosphorylated by phosphoglucan phosphatases SEX4 (Starch Excess) and LSF2 (LIKE SEX FOUR2), first described in A. thaliana. These processes are interrelated: phosphorylation by dikinases increases granule solubility and makes them accessible for amylases, whereas phosphate moieties may hamper hydrolysis (Hejazi et al., 2010; Santelia et al., 2011). Reduction of SEX4 or LSF2 activities in potato inhibited starch degradation in leaves. Starch content in tubers remained unchanged, and granules were smaller and less phosphorylated (Samodien et al., 2018).

The cooperation of dikinases, amylases, and phosphatases produces a pool of soluble maltooligosaccharides (linear glucans). Maltooligosaccharides are degraded by two pathways: hydrolytic, by β-amylases, or phosphorolytic, by α-glucan phosphorylase PHO1 (Weise et al., 2006; Fulton et al., 2008). The end product of the phosphorolytic pathway is G1P, which can be utilized in metabolism inside the plastid. Also, glucose can be produced by 4-α-glucanotransferase DPE1 (DisProportionating Enzyme) and exported to cytosol by glucose transporter pGlcT1 (Critchley et al., 2001; Cho et al., 2011). Knockdown of the chloroplast enzyme DPE slows down starch degradation in potato leaves in the cold and induces maltooligosaccharide accumulation, although these effects are not observed in tubers (Lloyd et al., 2004). Cold-induced sweetening in potato tubers is accompanied by increasing β-amylase activity and higher maltose content (Nielsen et al., 1997).

Maltose, which is the predominant product of hydrolytic starch degradation, is exported to cytosol by the transmembrane transporter MEX1 (Cho et al., 2011). In cytosol, maltose is processed by 4-α-glucanotransferase DPE2 or phosphorylase PHO2 to glucose or G1P, which are then converted to sucrose by the joint action of PGI2, PGM2, HK, UGPase, SPS (sucrose phosphate synthase), and SPP (sucrose phosphate phosphatase) (see the Figure) (López-González et al., 2019).

Sucrose is exported from leaf cells to storage organs; also, it is used in cell metabolism. In potato tubers, sucrose is used as a source of nutrients in sprouting, and its level controls dormancy release (Sonnewald S., Sonnewald U., 2014).

To delay sprouting, potato tubers are stored at low temperatures, 2–5 °C, and these conditions initiate cold-induced sweetening. This process involves sucrose hydrolysis by vacuolar acid invertase AcInv, encoded by the Pain-1 gene, and the accumulation of reducing sugars (glucose and fructose) in tubers (see the Figure) (Sowokinos et al., 2018). Knockout of Pain-1 resulted in lower contents of reducing sugars (Clasen et al., 2016). One of the key regulators of cold-induced sweetening is invertase inhibitor SbAI, which inhibits AcInv (McKenzie et al., 2013). It has been shown that SbAI can also inhibit α- and β-amylases (StAmy23, StBAM1, and StBAM9), in potato tubers, thereby influencing the rate of starch degradation in cold-induced sweetening (Zhang H. et al., 2014).

## Mechanisms controlling starch metabolism

Starch metabolism requires orchestrated work of many enzymes, transporters, and targeting proteins, which implies many regulation levels: gene expression, posttranscriptional regulation, and the posttranslational regulation of enzymatic activity. The expression patterns of genes for key enzymes involved in starch metabolism are well known in various plant species, but much less is known about expression-regulating factors (López- González et al., 2019). The difficulty is that the starchmetabolizing enzymes exist as numerous isoforms, which are encoded by the corresponding number of paralogous genes. The expression patterns of these genes depend on tissue (leaves, developing seeds, or growing tubers) and developmental stage, as shown in A. thaliana and maize (Tsai et al., 2009; Chen et al., 2014). In potato, the tissue-specific mode of expression has been shown for SuSy4, SS5, SBE3, APL3, PHO1a, PHO1b, GPT1.1, GPT2.1, SEX4, and NTT2 in tubers and for AMY1.1, APL1, and BAM3.1 in leaves (Van Harsselaar et al., 2017). A number of external and internal factors affect the expression of starch biosynthesis genes: circadian rhythms, photoperiod, and sugar content (Tiessen et al., 2002; Kötting et al., 2010). It is known that the expression rates of GBSSI, LSF1, LSF2, SEX4, and BAM3 in A. thaliana leaves are governed by transcription factors depending on circadian rhythms and photoperiod, so that the demand for energy is rapidly met in response to ambient changes (Tenorio et al., 2003; Flis et al., 2016). The expression rates of the genes GBSS, SuSy, and AGPase respond to photoperiod in growing potato tubers, being highest in the end of the light time and lowest in the beginning. This variation is determined by the influx of photoassimilates from leaves (Geigenberger, Stitt, 2000; Ferreira et al., 2010).

The formation of the storage organ, tuber, from the stolon is an important step in potato plant development. It includes intense starch production, the formation of starch granules, and increase in metabolite flux. Tuber formation is a complex process, influenced by environmental factors (photoperiod) and a variety of signals: biochemical, hormonal, and molecular, mediated by microRNAs and transcription factors (Hannapel et al., 2017; Kondhare et al., 2021). The investigation of tuber formation contributed much to the understanding of mechanisms that regulate starch metabolism in potato tubers.

Plant hormones are an important factor influencing the expression of genes involved in starch metabolism, and their effect on tuber formation has been studied in sufficient detail. The level of abscisic acid correlates with starch accumulation in potato tubers (Borzenkova, Borovkova, 2003). Treatment of stolons with indole acetic acid increased starch content in growing tubers, but a twofold increase in concentration caused the opposite effect (Wang D. et al., 2018). A correlation between the transcription rates of the genes for auxin, on the one hand, and starch biosynthesis (PGM, AGPase, GBSS, SS, and BE), on the other hand, was observed in the formation of cassava storage roots (Rüscher et al., 2021).

Sugars (hexoses, sucrose, and trehalose) are another group of signaling molecules influencing the expression of starch metabolism genes. Sucrose increases the expression of the SuSy and AGPase genes in potato (Salanoubat, Belliard, 1989; Müller-Röber et al., 1990). The rates of SuSy and AGPase expression are high in growing tubers, but they decrease rapidly after the separation of the tuber from the plant and, correspondingly, cease of sucrose import from photosynthesizing organs (Ferreira et al., 2010).

The differential expression of starch biosynthesis genes was detected at various tuber development stages (Ferreira et al., 2010; Van Harsselaar et al., 2017). The expression rate of the SS4 gene was elevated at the stolon stage, and it lowered with tuber growth, confirming the role of this starch synthase in granule initiation (Ferreira et al., 2010). Also, tuber growth was accompanied by an increase in the expression rate of sucrose synthase SuSy4 and decrease in the expression of cell wall invertase cw-Inv. These changes point to transition to the sucrose synthase-mediated pathway of sucrose degradation. Genes for glucose-6-phosphate translocator GPT, adenylate translocator NTT, ADP-glucose pyrophosphorylase (AGPase), starch synthases, and starch-branching enzymes increased their expression with tuber growth. Of this group, the isogenes SuSy4, SBE3, and GPT2.1 demonstrated just tuber-specific expression (Ferreira et al., 2010; Van Harsselaar et al., 2017). Coexpression analysis was employed to investigate the mechanisms of molecular regulation of gene activity, and transcription factors LOB, TIFY5a, and WRKY4 were found to be associated with the expression of the SuSy4 and GPT2.1 genes (Van Harsselaar et al., 2017). Analysis of coexpression networks for starch biosynthesis genes of seven plant species (Arabidopsis thaliana, cassava Manihot esculenta, millet Panicum virgatum, maize Zea mays, rice Oryza sativa, barley Hordeum vulgare, and sweet potato Ipomoea batatas) revealed the involvement of 24 transcription factors (López-González et al., 2019).

Little is known about mechanisms regulating starch metabolism genes at the posttranscriptional step. Posttranscriptional regulation involves a variety of factors, including RNA-binding proteins (RBPs), microRNAs, and alternative splicing, so that plants can rapidly reprogram their transcriptomes in response to external and internal factors. Photoperiod significantly influences microRNA expression patterns in the growth and development of potato tubers. It has been shown that differentially expressing microRNAs are targeted to genes coding for transcription factors and RNA-binding regulatory proteins StGRAS, StTCP2/4, and StPTB6 (Kondhare et al., 2018).

Posttranslational regulation is the next step of protein activity control. It is mediated by allosteric regulation, in which an effector molecule is bound to a noncatalytic site of the enzyme, altering its conformation, catalytic properties, and, thereby, its specificity and interaction with other proteins (Zeeman et al., 2010). Allosteric regulation involves protein phosphorylation and the formation of multimeric complexes and disulfide bridges (Kötting et al., 2010; Zeeman et al., 2010). Many starch-metabolizing enzymes assume the phosphorylated state: PGI, PGM1, AGPase, SS3, GWD1, GWD2, DPE2, AMY3, BAM1, BAM3, LDA, pGlcT, and MEX1 (Kötting et al., 2010). ADP-glucose pyrophosphorylase (AGPase) is a clear example of allosterically regulated potato enzyme. It is activated by 3-phosphoglyceric acid and inhibited by inorganic phosphate (Sowokinos, Preiss, 1982). Depending on the redox state in the cell, AGPase can be reversibly inactivated by the formation of disulfide bridges between small subunits of the heterotetramer (Ballicora et al., 2000).

Enzymes can aggregate into complexes known as metabolons (Sweetlove, Fernie, 2013). Complexes formed by starch biosynthesis enzymes were found in the endosperm of growing cereal seeds; in particular, SSIII, SSIIa, SBEIIa, and SBEIIb form a protein complex (Tetlow et al., 2008). Protein complexes of PTST2 and SS4 form in the initiation of starch granules in A. thaliana leaves (Seung et al., 2015, 2017). Potato isoamylases ISA1 and ISA2 form a heterotetrameric complex, which controls starch granule formation (Bustos et al., 2004).

## Conclusion

The investigation of starch metabolism in potato plants, particularly, starch biosynthesis and degradation in tubers, is topical in connection with the growing demand for potato starch in industry. A large body of information on key enzymes for starch and carbohydrate metabolism in various crops and the model species A. thaliana has been accumulated in the past three decades. Although the starch biosynthesis scheme is basically the same in different species, there are significant variations associated with different sets of isozymes, features of their functions, metabolite transport pathways (e. g., ADP-glucose transport through plastid membranes in cereals), and the existence of intricate and multileveled regulation, governed by external (photoperiod and temperature) and internal (plant hormones, metabolites, microRNA, and regulatory proteins) factors. Isogenes encoding six starch synthase isoforms, seven sucrose synthases, nine β-amylases, and three to five for each of the starch-branching and other enzymes were identified in the potato genome (Van Harsselaar et al., 2017).

The functions of many isoforms, including the majority of α- and β-amylases, are still unknown. Some isogenes (SuSy4, SS5, SBE3, APL3, PHO1a, PHO1b, GPT1.1, GPT2.1, SEX4, and NTT2) demonstrate tuberspecific expression and activity variation at various stages of tuber formation (Ferreira et al., 2010; Van Harsselaar et al., 2017). Isoenzymes AMY23, BAM1, BAM9 are specifically involved in starch degradation and carbohydrate metabolism in cold-induced sweetening (Hou et al., 2017). Also, the action of various factors on starch accumulation during tuber development has been shown: transcription factors LOB, TIFY5a, and WRKY4; plant hormones (auxin and abscisic acid); sugars; and microRNAs, the contents of which may mediate the effect of photoperiod. However, the functions of many isoenzymes and proteins involved in the regulatory and directing functions in starch metabolism in potato plants are poorly explored. To resolve this issue, modern methods are proposed: combined analysis of the metabolome and transcriptome inside a single cell or tissue (López-González et al., 2019). Bottom-up proteomics also seems promising in search for new components (Helle et al., 2018). For example, the analysis of 36 proteins associated with potato starch granules revealed, in addition to already known starch metabolism enzymes, targeting and regulatory proteins described in A. thaliana: PTST1 (Protein Targeting to Starch), ESV1 (Early StarVation1), and LESV (Like ESV). Also, Kunitz-type proteinase inhibitor and enzymes involved in redox regulation (thioredoxin TRX and glutathione peroxidase GPX) were found (Helle et al., 2018). Detailed information on all components involved in starch metabolism and on their interactions, including their behavior under varying ambient conditions, is essential for raising potato varieties with high performance and specified starch properties.

## Conflict of interest

The authors declare no conflict of interest.
